# Dysuria after endoscopic laser enucleation of the prostate: how common, how long, and which patients are at risk? A prospective cohort analysis

**DOI:** 10.1007/s00345-026-06556-1

**Published:** 2026-06-23

**Authors:** Oswaldo Oliveira Neto, Gabriel Eufrasio da Silva, Henri Luiz Morgan, Marcelo Petan Amaro, Ricardo Haidar Berjeaut, Victor Srougi, Miguel Srougi, William Carlos Nahas, Alberto Azoubel Antunes

**Affiliations:** University of São Paulo School of Medicine, São Paulo, Brazil

**Keywords:** Dysuria, Endoscopic enucleation of the prostate, Benign prostatic hyperplasia (BPH), Holmium laser enucleation of the prostate (HoLEP), Lower urinary tract symptoms (LUTS), Postoperative recovery

## Abstract

**Purpose:**

Postoperative dysuria after endoscopic laser enucleation of the prostate (EEP) is common, yet its true incidence, temporal profile, and determinants remain poorly defined. We aimed to characterize the incidence, severity, and predictors of moderate-to-severe dysuria up to 6 months after EEP.

**Methods:**

In this prospective cohort study, we analyzed 108 patients who underwent EEP using Holmium (standard or Moses^®^) or pulsed Thulium (RealPulse^®^) lasers. Dysuria was assessed weekly using a Likert scale and categorized as mild (1–2), moderate (3–7), and severe (8–10). Patients with moderate-to-severe dysuria (score ≥ 3) were grouped for subsequent analyses. Associations were evaluated using Spearman correlation and multivariable logistic regression.

**Results:**

Moderate-to-severe dysuria (score ≥ 3) was highly prevalent early after surgery, affecting 75.9% of patients at week 1 and 29.6% at week 4, but declined sharply thereafter (8.3% at 3 months and 0.9% at 6 months). Early dysuria was associated with younger age, diabetes, and clot retention. Between 1 and 3 months, dysuria showed weak associations, with operative and functional variables, including operative time, total energy, enucleation efficiency, bipolar energy use, prior prostate biopsy, and postoperative Qmax. On exploratory multivariate analysis, younger age independently predicted early dysuria (OR 0.90, 95% CI 0.84–0.97, *p* = 0.007), while prior prostate biopsy predicted dysuria between 1 and 3 months (OR 10.85, 95% CI 1.12–105.15, *p* = 0.040).

**Conclusions:**

Postoperative dysuria after EEP is highly prevalent during the early postoperative period but follows a predominantly self-limited course, with near-complete resolution after 12 weeks. These findings provide a detailed characterization of symptom evolution over time and may help improve postoperative counseling and patient expectations regarding recovery. Exploratory analyses identified associations with younger age and prior prostate biopsy.

## Introduction

Benign prostatic hyperplasia (BPH) is the most common cause of lower urinary tract symptoms (LUTS) in aging men, affecting approximately 50% of men over 50 years of age and up to 90% in the ninth decade of life in autopsy studies [1]. LUTS include storage and voiding symptoms and may significantly impair quality of life, in addition to being associated with complications such as recurrent urinary tract infections, hematuria, acute and chronic urinary retention, and renal dysfunction [2, 3].

Surgical treatment is indicated in cases of treatment failure, intolerance to medical therapy, or development of complications. Endoscopic laser enucleation of the prostate (EEP) has emerged as a first-line surgical option and has been endorsed by international guidelines independent of prostate size with durable functional outcomes [4, 5]. EEP has demonstrated superior perioperative outcomes compared with transurethral resection, including reduced bleeding, shorter catheterization, and shorter hospital stay [6, 7].

Despite the advantages, dysuria is a frequently reported postoperative complaint after endoscopic prostate surgery. According to the International Continence Society, dysuria refers to pain, burning, or discomfort during voiding and is often transient in the postoperative setting [8]. Previous studies have reported dysuria in up to 28–33% of patients during the first months after HoLEP [9, 10].

Technological advances, including pulse-modulated Holmium systems (MOSES^®^) and the introduction of pulsed solid-state Thulium lasers (p-Tm: YAG), have aimed to improve enucleation efficiency and hemostasis [11–14]. However, clinical data comparing different laser technologies with respect to postoperative dysuria remain scarce, and patient- and procedure-related risk factors for this symptom are poorly defined.

The objective of this study was to evaluate the incidence, severity, and temporal pattern of dysuria after endoscopic laser enucleation of the prostate, and to identify preoperative, intraoperative, and postoperative factors associated with its occurrence.

## Materials and methods

This prospective longitudinal cohort study evaluated consecutive patients undergoing EEP for benign prostatic obstruction at a tertiary referral center between 2022 and 2024.

Eligible patients were men diagnosed with BPH and scheduled for EEP. Exclusion criteria included suspicion of prostate malignancy, cognitive impairment precluding reliable symptom reporting, prior pelvic surgery with bladder dysfunction, previous pelvic radiotherapy, chronic pelvic pain syndrome, and refusal to provide informed consent.

All procedures were performed transurethrally under spinal or general anesthesia by experienced surgeons using holmium laser (Standard or with Moses^®^ technology) or pulsed thulium laser (RealPulse^®^ technology), according to availability. Enucleation followed standardized anatomical principles, predominantly using an *en bloc* technique, with morcellation performed in all cases. Adjunctive bipolar coagulation for hemostasis was employed at the end of selected procedures.

Dysuria was prospectively assessed by actively questioning patients about pain or burning during voiding using a numeric Likert scale ranging from 0 to 10. Assessments were performed at predefined postoperative intervals: weeks 1, 2, 3, 4, and 6, followed by monthly assessments up to 6 months. For analysis according to symptom intensity, dysuria scores were categorized as mild (scores 1–2), moderate (scores 3–7), and severe (scores 8–10). A score of 0 was considered absence of dysuria. Patients with moderate or severe dysuria (score ≥ 3) were grouped for subsequent analyses. This threshold was predefined to distinguish symptoms beyond mild discomfort and to facilitate characterization of postoperative symptom burden and its temporal evolution during recovery.

Patients with persistent dysuria underwent urinalysis and urine culture. Cases with confirmed postoperative urinary tract infection were excluded from all dysuria-related analyses, as symptoms in these patients could not be reliably distinguished from infection-related urinary discomfort.

Demographic data, preoperative clinical variables, perioperative parameters, postoperative complications, and functional outcomes were prospectively recorded. Associations between dysuria severity and clinical variables were analyzed separately for the first postoperative month and for the period between 1 and 3 months.

Statistical analyses were performed using SPSS software (IBM SPSS Statistics, version 22.0). Continuous variables were expressed as mean and standard deviation, and categorical variables as absolute numbers and percentages. Correlations were assessed using Spearman’s rank correlation coefficient. Exploratory multivariable logistic regression analyses were performed to evaluate factors associated with moderate-to-severe dysuria (score ≥ 3) at postoperative week 1 and week 6. Variables demonstrating statistically significant associations in univariable analyses (*p* < 0.05) were entered into the multivariable models. A p value < 0.05 was considered statistically significant.

The study was approved by the local Ethics Committee and conducted in accordance with the Declaration of Helsinki. Written informed consent was obtained from all participants.

## Results

A total of 138 patients were screened, and 108 were included in the final analysis after application of exclusion criteria and confirmation of complete follow-up. Mean age was 69.3 years (SD 7.27). Mean prostate volume was 98.6 mL (SD 46.3). Preoperative maximum urinary flow rate (Qmax) was 9.8 mL/s (SD 3.92). Lower urinary tract symptoms were severe at baseline, with a mean IPSS of 25.7 (SD 5.34) and a mean quality-of-life score of 5.17 (SD 0.88). Diabetes mellitus was present in 20.4% of patients.

Mean enucleation and morcellation times were 74.2 min (SD 34) and 12.1 min (SD 11.1), respectively, with a mean enucleated tissue weight of 55.2 g (SD 32.8). Mean enucleation efficiency was 0.75 g/min (SD 0.56), and morcellation efficiency was 5.31 g/min (SD 3.26). The mean total laser energy delivered was 197 kJ (SD 122). Adjunctive bipolar resection loop for hemostasis was used in 38% of cases. Mean total operative time, including enucleation and morcellation, was 96.4 min (SD 41.4). Laser technologies included HoLEP MOSES (29.6%), pulsed ThuLEP (32.4%), and standard HoLEP (38.0%).

Postoperative outcomes improved substantially over time. Mean IPSS decreased to 8.36 (SD 6.0) at 1 month, 4.57 (SD 4.83) at 3 months, and 2.62 (SD 4.54) at 6 months. Quality-of-life scores improved accordingly, and postoperative Qmax increased to 24.9 mL/s (SD 11.4) (Table 1).

**Fig. 1:**
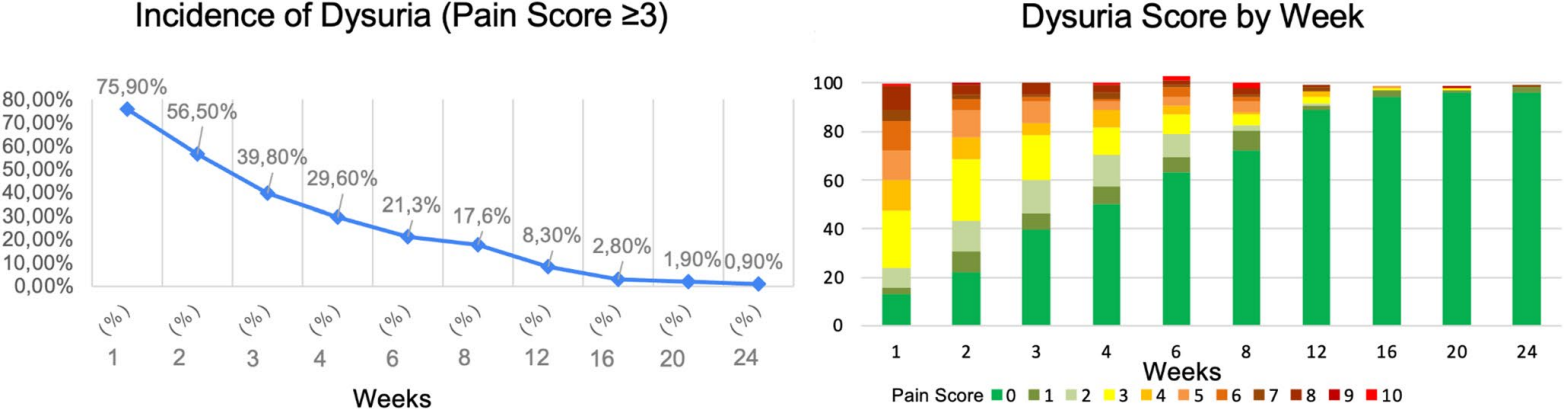
Incidence (**a**) and severity (**b**) of postoperative dysuria

The incidence of moderate-to-severe dysuria (score ≥ 3) was 75.9% in the first postoperative week. This proportion decreased to 56.5% in the second week and 39.8% in the third week. A continued reduction was observed during follow-up, with moderate-to-severe dysuria reported by 29.6% of patients at week 4, 21.3% at week 6, and 17.6% at week 8. From week 12 onward, moderate-to-severe dysuria became infrequent, occurring in 8.3% of patients at week 12, 2.8% at week 16, 1.9% at week 20, and 0.9% at week 24 (Fig. 1a and b).

During the first postoperative month, dysuria was correlated with younger age, diabetes mellitus, and clot retention. On multivariate analysis, only younger age remained independently associated with moderate-to-severe dysuria in the first postoperative week (OR 0.90, 95% CI 0.84–0.97, *p* = 0.007) (Table 2).

Between the first and third postoperative months, dysuria was associated with procedural and patient-related factors, including operative time, enucleation time, total energy, enucleation efficiency, bipolar energy use, prior prostate biopsy, and postoperative Qmax. No significant associations were observed with PSA, prostate volume, preoperative catheterization, urine culture, or laser type.

On exploratory multivariable analysis, a history of prostate biopsy was independently associated with moderate-to-severe dysuria at 6 weeks (OR 10.85, 95% CI 1.12–105.15, *p* = 0.040), while other variables were not independently associated after adjustment (Table 2).

## Discussion

To our knowledge, this is the first prospective study to systematically characterize the incidence, severity, and temporal evolution of dysuria following EEP during the earliest postoperative weeks. The principal finding of this study is that postoperative dysuria is highly prevalent during the early recovery period, affecting approximately three-quarters of patients during the first postoperative week when moderate-to-severe symptoms (score ≥ 3) were used as the predefined threshold. However, symptom burden progressively declined over time, reaching 8.3% by 12 weeks and becoming nearly absent by 6 months. These findings support the concept of dysuria as a predominantly transient and self-limited postoperative symptom and provide a detailed description of its natural history following EEP.

The reported incidence of dysuria after transurethral prostate surgery varies widely across studies, largely due to heterogeneity in symptom definitions, assessment methods, and follow-up schedules. In a recent systematic review by Wroclawski et al. [15], the pooled incidence of postoperative pelvic pain—including dysuria—was estimated at 9% within the first postoperative month. However, substantial heterogeneity and the limited availability of longitudinal data restrict interpretation of these findings. In contrast, the prospective design and repeated assessments used in the present study provide a more detailed and clinically relevant characterization of symptom severity and recovery over time.

The clinical relevance of these findings extends beyond symptom quantification alone. Dysuria is among the most common concerns reported during recovery after EEP and may contribute substantially to postoperative anxiety when its expected course is not adequately communicated. Previous studies have demonstrated that improving preoperative patient education regarding postoperative dysuria significantly increases patient understanding of recovery expectations, with most patients reporting symptom severity that was equal to or less than anticipated after surgery [16]. Therefore, a detailed understanding of the incidence, severity, and temporal course of postoperative dysuria may help optimize preoperative counseling, establish realistic recovery expectations, and improve the overall patient experience following EEP.

An important observation from the present study is that recovery after EEP should not be viewed solely through improvements in objective functional outcomes. Although relief of bladder outlet obstruction is often achieved immediately after surgery, irritative symptoms such as dysuria may follow a distinct recovery trajectory. Consequently, patients may experience satisfactory urinary flow and voiding function while still reporting discomfort during the early postoperative period. This distinction has practical implications for postoperative management. While dysuria was highly prevalent during the first weeks after surgery, its progressive spontaneous resolution suggests that symptom persistence during the early recovery period should not necessarily be interpreted as evidence of postoperative complications. Rather, in the absence of other concerning findings, these symptoms may represent part of the expected inflammatory healing process following EEP.

Dysuria demonstrated weak but statistically significant associations with several patient- and procedure-related variables. The modest magnitude of these correlations suggests that postoperative dysuria is unlikely to be explained by a single determinant and instead reflects a multifactorial process involving patient characteristics, perioperative factors, and postoperative recovery. Consequently, the identified associations should be interpreted as exploratory rather than indicative of a dominant underlying mechanism.

Exploratory multivariable analysis suggested a temporal shift in factors associated with dysuria. Younger age was independently associated with early postoperative dysuria, whereas prior prostate biopsy was associated with dysuria between 1 and 3 months. Although the biological basis of these findings remains uncertain, younger patients may perceive or report postoperative discomfort differently from older individuals [17, 18]. Similarly, prior prostate biopsy could plausibly reflect pre-existing inflammatory alterations within the prostatic microenvironment [17, 19–21]. However, these interpretations remain speculative, as the present study was not designed to investigate underlying mechanisms.

Several additional variables, including diabetes, clot retention, operative time, enucleation time, total energy use, enucleation efficiency, and postoperative urinary flow rate, demonstrated weak associations with dysuria in univariable analyses. However, these associations were inconsistent across follow-up periods and generally not maintained after multivariable adjustment. Given their modest magnitude and the observational nature of the study, these findings should be regarded as hypothesis-generating and require confirmation in larger prospective cohorts.

No association was observed between laser platform and dysuria, suggesting that this symptom is driven predominantly by patient-related and inflammatory or technical factors rather than by the specific energy source.

This study has several limitations. Its observational design precludes causal inference, although prospective data collection and consecutive patient inclusion help mitigate selection bias. Dysuria was assessed using a numeric patient-reported symptom scale that has not been specifically validated for postoperative dysuria following EEP; therefore, the symptom severity categorization used in this study should be interpreted as a predefined symptom severity classification developed for the purposes of this study. In addition, psychosocial factors that may influence symptom perception were not assessed. Finally, the association analyses should be interpreted cautiously given the sample size, the number of variables examined, and the limited number of events during later follow-up periods. Consequently, the identified associations should be regarded as exploratory and hypothesis-generating rather than definitive predictors of postoperative dysuria.

Nevertheless, the prospective design, systematic symptom assessment, repeated early postoperative evaluations, and complete follow-up strengthen the validity of the present findings. By providing a detailed characterization of the incidence, severity, and temporal evolution of dysuria after EEP, this study contributes to a better understanding of a common but incompletely described postoperative symptom. These data may help improve patient counseling, establish realistic expectations regarding recovery, and support more informed postoperative management.

## Conclusion

Postoperative dysuria after EEP is a frequent but predominantly transient symptom. Although approximately three-quarters of patients experience moderate-to-severe dysuria during the first postoperative week, symptoms progressively improve and become uncommon after 12 weeks. This prospective characterization of symptom evolution may assist clinicians in counseling patients and establishing realistic expectations regarding postoperative recovery. Exploratory analyses suggest associations between postoperative dysuria, younger age, and prior prostate biopsy, generating hypotheses for future investigation.

**Table 1:** Preoperative, intraoperative and postoperative characteristics

Variable	Total (*n* = 108)
*Preoperative*	
Age, mean (SD)	69.3 (7.27)
Prostate volume, mL, mean (SD)	98.6 (46.3)
Postvoid residual volume, mL, mean (SD)	128 (259)
Indwelling urinary catheter, n (%)	21 (19.8)
Previous prostate biopsy, n (%)	43 (41.3)
PSA, ng/mL, mean (SD)	5.86 (4.25)
Pretoperative Qmax – mean (SD)	9.8 (3.92)
IPSS, mean (SD)	25.7 (5.34)
Quality of life score (QoL), mean (SD)	5.17 (0.88)
Positive preoperative urine culture, n (%)	18 (18.5)
*Intraoperative*	
Enucleation time, min – mean (SD)	74.2 (34.0)
Morcellation time, min – mean (SD)	12.1 (11.1)
Enucleated tissue weight, g – mean (SD)	55.2 (32.8)
Enucleation efficiency, g/min – mean (SD)	0.75 (0.56)
Morcellation efficiency, g/min – mean (SD)	5.31 (3.26)
Total laser energy delivered, kJ – mean (SD)	197 (122)
Adjunctive bipolar loop for hemostasis, n (%)	41 (38%)
HoLEP MOSES, n (%)	32 (29.6%)
Pulsed ThuLEP, n (%)	35 (32.4%)
Conventional HoLEP, n (%)	41 (38.0%)
*Postoperative*	
PSA, ng/mL, mean (SD)	1.03 (0.98)
Postoperative Qmax – mean (SD)	24.9 (11.4)
Prostate volume, mL, mean (SD)	34 (11.3)
Catheterization time (h) – median (SD)	24 (30.3)
IPSS at 1 month – mean (SD)	8.36 (6.0)
QoL at 1 month – mean (SD)	1.94 (1.31)
IPSS at 3 months – mean (SD)	4.57 (4.83)
QoL at 3 months – mean (SD)	1.07 (1.26)
IPSS at 6 months – mean (SD)	2.62 (4.54)
QoL at 6 months – mean (SD)	0.53 (0.97)

**Table 2:** Multivariate analysis of variables associated with moderate/severe dysuria after EEP

Period	Variable	*r* (Spearman)	*p*-value	OR (multivariable)	95% CI	*p*-value
≤ 1 month	Age	–0.30	0.002	**0.90**	**0.84–0.97**	**0.007**
	Diabetes	+ 0.21	0.029	–	–	–
	Clot retention	+ 0.26	0.011	–	–	–
1–3 months	History of prostate biopsy	+ 0.32	0.001	**10.85**	**1.12–105.15**	**0.040**
	Operative time	–0.20	0.037	–	–	–
	Enucleation time	–0.20	0.041	–	–	–
	Enucleation efficiency	–0.21	0.026	–	–	–
	Total energy used	–0.29	0.004	0.99	0.98–1.00	0.064
	Bipolar energy use	+ 0.19	0.048	0.11	0.01–1.38	0.088
	Postoperative Qmax	–0.35	0.001	0.92	0.84–1.02	0.100
	Other variables*	–	≥ 0.05	–	–	–

*Variables tested with no significant association included preoperative PSA, prostate volume, preoperative catheterization, preoperative positive urine culture, laser type, hypertension, BPH/LUTS symptom duration, prior urinary retention, anticoagulant use, and energy density. Bold values indicate statistically significant results in multivariable analysis (p < 0.05)

## Data Availability

No datasets were generated or analysed during the current study.
